# From Meshes to Minimally Invasive Techniques: A Comprehensive Review of Modern Hernia Repair Approaches

**DOI:** 10.7759/cureus.66206

**Published:** 2024-08-05

**Authors:** Akansha Hatewar, Chanrashekhar Mahakalkar, Shivani Kshirsagar, Poosarla Ram Sohan, Sparsh Dixit, Shruthi Bikkumalla

**Affiliations:** 1 General Surgery, Jawaharlal Nehru Medical College, Datta Meghe Institute of Higher Education and Research, Wardha, IND

**Keywords:** biological meshes, robotic-assisted surgery, laparoscopic surgery, minimally invasive surgery, mesh repair, hernia

## Abstract

Hernias are a common medical condition characterized by the protrusion of organs or tissues through weakened muscle walls, affecting millions worldwide annually. Historically, from being treated with open surgeries using tension-free mesh repairs, the landscape of hernia repair has evolved significantly. This evolution has been marked by the advent and refinement of minimally invasive techniques, including laparoscopic and robotic-assisted approaches, which offer reduced postoperative pain, shorter recovery times, and improved patient outcomes compared to traditional methods. This comprehensive review aims to elucidate the evolution of hernia repair techniques, emphasizing the transition from conventional mesh repairs to advanced minimally invasive methodologies. By examining the historical progression and current state of hernia surgery, this review thoroughly analyzes the advancements in surgical techniques, materials, and technologies. Furthermore, it explores emerging trends such as biological meshes, ultrasound-guided procedures, and 3D printing applications in hernia repair. The clinical significance of these advancements lies in their potential to enhance the patient's quality of life, minimize complications, and optimize healthcare resource utilization. Insights gained from this review will inform clinicians and researchers about the efficacy, safety, and comparative effectiveness of various hernia repair approaches, guiding future directions in hernia management and fostering innovation in surgical practice.

## Introduction and background

Hernias represent a prevalent and significant health concern worldwide, characterized by the protrusion of an organ or tissue through an abnormal opening in the muscle wall [1]. They can occur in various anatomical locations, with inguinal, femoral, umbilical, and incisional hernias being among the most common types observed clinically. Globally, hernias affect millions of individuals annually, posing challenges to healthcare systems and necessitating effective treatment strategies [1]. The treatment landscape for hernias has evolved significantly over the decades, transitioning from traditional open surgeries with tension-free mesh repairs to minimally invasive techniques that offer reduced recovery times and improved patient outcomes. This evolution has been driven by advancements in surgical technology, techniques, and materials aimed at enhancing patient comfort, reducing complication rates, and achieving better long-term results [2].

The importance of these advancements in hernia repair cannot be overstated. Improved surgical outcomes contribute to better patient quality of life, reduced healthcare costs, and shorter hospital stays. Minimally invasive approaches, such as laparoscopic and robotic-assisted surgeries, have revolutionized hernia repair by minimizing trauma to surrounding tissues, decreasing postoperative pain, and accelerating recovery periods [3]. This review aims to comprehensively explore the evolution of hernia repair techniques, from traditional mesh repairs to the latest minimally invasive approaches. By examining the historical progression, current methodologies, and emerging innovations in hernia surgery, this review seeks to provide clinicians and researchers with a detailed understanding of the advancements in the field. Additionally, it aims to highlight the clinical significance of these advancements and their implications for improving patient outcomes and quality of life.

## Review

Traditional mesh repair techniques

Overview of Tension-Free Repairs

The Lichtenstein tension-free mesh repair technique employs an open anterior approach, positioning a mesh patch over the hernia defect on the front of the abdominal wall muscles. This method "circumvents the challenge of working with degenerated tissue by placing the patch's edges on surrounding healthy tissue, thus providing more robust reinforcement for the abdominal wall." Research indicates that the Lichtenstein technique has a low recurrence rate of approximately 2% [4]. Another option is the Prolene® Hernia System (PHS), a "three-in-one" device with an onlay patch, a connector piece, and an underlay patch. It reinforces the area behind the abdominal wall and covers the entire hernia-prone myopectineal orifice. Proponents argue that it integrates the advantages of the Lichtenstein, mesh plug, and Kugel techniques [4]. The Shouldice Hospital in Canada has conducted over 250,000 hernia repairs using the Shouldice technique, a "pure tissue" tension repair method that employs steel wire sutures. They report very low recurrence rates with this approach, which eschews the use of mesh [5]. The British Hernia Centre technique involves placing a fine mesh at the hernia defect without stitching the muscle tissue. The mesh is securely held in place, and the muscle or tendon grows around and through it during healing, offering complete, tension-free abdominal wall reinforcement [6]. The primary benefits of tension-free mesh repairs include extremely low recurrence rates, often below 2%, minimal postoperative pain, rapid recovery, and comprehensive reinforcement of the hernia-prone area. While traditional tension repairs avoid mesh, they have higher recurrence rates. Consequently, modern tension-free mesh techniques have become the gold standard for hernia repair in most cases [7].

Types of Meshes Used

Synthetic meshes are the most commonly utilized materials for hernia repair, representing 90-95% of all procedures. Among these, polypropylene mesh is the most prevalent, promoting tissue ingrowth but potentially leading to complications such as chronic infection, fistulas, and erosion over time. Polyester mesh, frequently used in Europe, offers strength and durability. Expanded polytetrafluoroethylene (ePTFE; Gore-Tex®) mesh is known for its minimal inflammatory reaction, though it inhibits tissue ingrowth, creating a barrier [8]. Composite meshes have been developed to mitigate the risk of adhesion formation when a mesh is placed in the peritoneal cavity. These meshes combine different materials to balance tissue ingrowth with adhesion prevention. For example, polypropylene/ePTFE composite mesh features a polypropylene side that supports tissue ingrowth and an ePTFE side that prevents adhesions. Another variant, oxidized regenerated cellulose-coated polypropylene mesh, incorporates a coating that acts as an adhesion barrier. However, no single composite mesh has demonstrated superior efficacy in preventing adhesions [9]. Biologic meshes, derived from animal tissues such as porcine, bovine dermis, or intestines, offer an alternative, especially in contaminated fields or when a permanent synthetic mesh is unsuitable. Acellular dermal matrix, obtained from human or animal skin, is used in infected fields, though its effectiveness in routine hernia repair remains under evaluation. Porcine small intestinal submucosa has also been employed, but a meta-analysis indicated that polytetrafluoroethylene (PTFE) was more effective for patch repair in congenital diaphragmatic hernia cases [10]. Selecting the appropriate mesh for hernia repair depends on various factors, including the type and severity of the hernia, patient characteristics, and the surgeon's expertise. Proper mesh choice and placement are crucial for successful hernia repair and minimizing complications [11].

Complications Associated With Traditional Approaches

The primary complications associated with traditional mesh repair techniques for hernias include infection, chronic pain, adhesions, mesh erosion and fistulization, and mesh failure. Infection is a notable concern as mesh repair techniques generally carry a higher infection risk than non-mesh repairs. Infection rates are reported to be between 6-10% for open ventral incisional hernia repairs with mesh. Factors contributing to mesh infection include obesity, chronic obstructive pulmonary disease, prior surgical site infections, prolonged operative times, and insufficient tissue coverage over the mesh [12]. Chronic pain is another frequent complication of mesh hernia repair. This pain can arise from nerve injury during surgery or nerve entrapment within the inflammatory response triggered by the mesh [13]. Additionally, mesh repair can lead to the formation of adhesions, which may result in intestinal obstruction. Computed tomography (CT) imaging is often employed to diagnose adhesions [14]. Mesh erosion into adjacent solid organs, such as the bladder or bowel, can cause fistulizing disease, a condition frequently identified through CT imaging. This complication can be particularly challenging for patients [15]. Finally, mesh failure, including shrinkage, detachment, and migration, can lead to hernia recurrence. Mesh failure is a common complication, with recurrence rates reported at 32% for mesh repairs compared to 63% for suture repairs alone [16].

Advancements in minimally invasive techniques

Introduction to Laparoscopic Hernia Repair

Laparoscopic hernia repair is a minimally invasive surgical approach to address various hernias, including inguinal, femoral, umbilical, incisional, and hiatal hernias. This technique involves making small incisions in the abdomen to insert a laparoscope - a thin, lighted instrument with an attached camera - and other surgical tools to perform the repair. The surgeon identifies the hernia defect, prepares the inner lining of the abdomen to support the mesh, and introduces a piece of mesh through one of the small incisions. The mesh is then stapled to cover and reinforce the weakened area [17]. Laparoscopic hernia repair is often recommended for patients with recurrent or bilateral hernias or those seeking a quicker return to normal activities. The procedure is typically performed under general anesthesia and may necessitate a hospital stay, depending on the hernia's type and complexity. The two most commonly employed laparoscopic techniques for inguinal hernia repair are the transabdominal preperitoneal (TAPP) repair and the extraperitoneal (TEP) repair. Both techniques aim to position the mesh in the optimal anatomical space, close the hernia defect, and minimize traumatic fixation [18]. Laparoscopic hernia repair offers several advantages over open surgery, including reduced postoperative pain, smaller incisions, faster recovery, and an earlier return to work. However, it may not be appropriate for patients with very large hernias, strangulated hernias, prior pelvic surgery, or those unable to tolerate general anesthesia. Despite these limitations, laparoscopic hernia repair remains a popular and effective method for treating hernias, offering a less invasive and less traumatic alternative to traditional open surgery [19].

Types of Laparoscopic Approaches

Minimally invasive surgical techniques have transformed hernia repair by providing patients with less invasive options and improved outcomes. Among these techniques, TAPP repair and TEP repair are two of the most commonly used laparoscopic approaches [20]. The TAPP approach involves accessing the peritoneal cavity, where a mesh is placed through a peritoneal incision into the preperitoneal space. The peritoneum is then closed over the mesh, positioning the mesh between the preperitoneal space and the peritoneum. This technique offers a comprehensive view of the myopectineal orifice, preventing unsuspected contralateral hernias [20]. In contrast, the TEP repair is conducted entirely within the preperitoneal space, avoiding entry into the abdominal cavity. This method is particularly beneficial for patients with a history of abdominal surgery as it bypasses the need to access the peritoneal cavity. Additionally, TEP can be performed without electrocautery, potentially resulting in reduced postoperative pain. During TEP, the preperitoneal space is developed, allowing exploration of three-quarters of potential hernia sites (femoral, obturator, and direct) on the contralateral side [21]. Both TAPP and TEP are well-established minimally invasive techniques for inguinal hernia repair. No definitive evidence indicates that one approach is superior to the other. The choice between TAPP and TEP often depends on the surgeon’s experience, preference, and specific patient factors [21].

Benefits Over Traditional Open Surgery (Reduced Pain, Faster Recovery)

The benefits of minimally invasive surgery compared to traditional open procedures are significant and extensive. One of the primary advantages is reduced pain and faster recovery times for patients. Minimally invasive techniques, which involve smaller incisions, cause considerably less trauma to the body than open surgery. As a result, patients experience markedly less postoperative pain and require fewer pain medications. They can also return to normal activities and resume work more quickly after a minimally invasive procedure [22]. In addition to enhancing the patient experience, minimally invasive surgeries often lead to shorter hospital stays. The smaller incisions and reduced tissue damage typically allow for earlier discharge from the hospital compared to open procedures. This not only benefits patients but also helps to reduce healthcare costs and resource utilization [3]. Another significant advantage of minimally invasive techniques is the improved cosmetic outcome. The small incisions result in minimal scarring, which is particularly beneficial for procedures performed in visible areas. This can positively affect a patient's self-esteem and overall satisfaction with the surgical results [23]. Finally, minimally invasive surgery often provides surgeons with better visualization and precision during the procedure. Using specialized instruments and advanced imaging technologies facilitates more accurate and effective treatment, improving patient outcomes. This enhanced accuracy is particularly valuable in complex or delicate procedures [24].

Comparative Effectiveness Studies

Minimally invasive surgical techniques have increasingly become the standard in hernia repair, providing patients with a less invasive alternative to traditional open procedures. Comparative effectiveness studies have highlighted the benefits and limitations of these innovative methods [11]. One area of focus has been comparing TAPP and enhanced view extraperitoneal (eTEP) techniques for ventral hernia repair. Both techniques offer advantages such as optimal mesh placement, effective closure of the hernia defect, and avoidance of traumatic fixation. A systematic review and meta-analysis found no significant differences between TAPP and eTEP regarding major complications, surgical site infection, seroma, recurrence, and other critical outcomes [25]. Another topic of discussion is the comparison between minimally invasive intraperitoneal onlay mesh (IPOM) repair and the traditionally recommended retromuscular mesh placement. Historically, guidelines have favored the retromuscular approach due to concerns about mesh-related complications. However, a recent study has challenged this view suggesting that minimally invasive IPOM repair remains a safe and effective option, especially for small to medium-sized hernias. This study reported a low incidence of long-term mesh-related complications and recommended reconsidering the avoidance of IPOM repairs based on concerns about severe complications [26]. Additionally, researchers have investigated the comparative effectiveness of hybrid hernia repair and laparoscopic hernia repair for incisional ventral hernias. A meta-analysis indicated that hybrid hernia repair has a significantly lower risk of seroma but a higher risk of wound infection than laparoscopic hernia repair. Nevertheless, no significant differences were found between the two techniques concerning operation time, blood loss, intestinal injury, intestinal obstruction, postoperative pain, mesh bulging, and recurrence [27].

Robotic-assisted hernia repair

Overview of Robotic Surgery in Hernia Repair

Robotic-assisted hernia repair techniques have gained traction in recent years due to their potential benefits over traditional open and laparoscopic methods. These robotic techniques include TAPP repair, TEP repair, and eTEP repair. Robotic TAPP involves making small abdominal incisions to access the preperitoneal space and placing a mesh to cover the hernia defect. Research indicates that robotic TAPP is associated with lower rates of chronic pain and recurrence compared to open hernia repair [28]. Robotic TEP, similar to robotic TAPP, also accesses the preperitoneal space without entering the peritoneal cavity. This approach eliminates the need for an abdominal incision and is linked to faster recovery times. Outcomes of robotic TEP regarding recurrence and chronic pain are comparable to those of robotic TAPP. Robotic eTEP offers an enhanced view of the surgical anatomy, improving the preperitoneal space's visualization and dissection. Early studies suggest no significant differences in major complications between robotic eTEP and traditional TAPP repairs [29]. The advantages of robotic-assisted hernia repair include enhanced precision and dexterity for complex dissection and mesh placement, improved visualization of the surgical anatomy, smaller incisions leading to reduced pain and quicker recovery, a lower risk of complications such as infection and hernia recurrence, and better ergonomics with reduced surgeon fatigue. Despite these benefits, robotic-assisted hernia repair currently represents less than 5% of all hernia repairs in Nordic countries. However, its adoption is rising as more robotic platforms are introduced [3]. For example, a center in Helsinki reported an increase in the rate of minimally invasive major ventral hernia surgeries from 25% to 75% following the implementation of a dedicated robotic hernia program. There is considerable potential for broader adoption of robotic hernia repair techniques, which could benefit over 50% of patients undergoing open hernia repair and nearly all those receiving laparoscopic repair [30].

Advantages and Disadvantages Compared to Laparoscopy

The robotic-assisted approach to hernia repair provides several key advantages over traditional laparoscopic techniques. Robotic arms offer enhanced maneuverability and precision compared to standard laparoscopic instruments, allowing for more intricate dissection and precise mesh placement. This increased dexterity can potentially lead to improved patient outcomes. Additionally, robotic systems deliver a high-definition 3D view of the surgical site, offering superior visualization of the anatomy and aiding in the precision of the procedure [31]. A notable benefit of robotic hernia repair is the reduction in pain and the faster recovery experienced by patients. As a minimally invasive procedure, robotic surgery results in smaller incisions and less tissue trauma than open repair. This often leads to decreased postoperative discomfort and a quicker return to normal activities. Robotic platforms' improved precision and visualization may also help lower the risk of complications, such as tissue damage and hernia recurrence [3]. However, robotic-assisted hernia repair does have some potential drawbacks. The primary concern is the higher cost associated with robotic surgical systems, which are more expensive to acquire and maintain than traditional laparoscopic equipment. Robotic procedures can also be more time-consuming than standard laparoscopic techniques, potentially affecting operating room efficiency and utilization. Surgeons need specialized training to become proficient in robotic-assisted techniques, which may limit the widespread adoption of this approach. Moreover, in complex cases involving significant scarring or anatomical challenges, robotic procedures may sometimes need to be converted to open surgery [32].

Clinical Outcomes and Evidence-Supporting Robotic Approaches

Robotic-assisted hernia repair has demonstrated several clinical benefits over traditional open and laparoscopic methods. Patients undergoing robotic-assisted ventral hernia repair generally experience a significantly shorter hospital stay than open repair. The average stay for robotic-assisted repair is approximately 0.5 days, whereas open repair typically results in a 2.1-day stay. Additionally, the incidence of readmission within 90 days post-discharge is notably lower for robotic-assisted repair, with a readmission rate of 12.1% compared to higher rates associated with open repair [33]. Robotic-assisted hernia repair is also associated with fewer complications, including a reduced risk of surgical-site issues, which are critical for minimizing the risk of hernia recurrence. Patients benefit from a faster recovery, with less pain and a shorter duration of postoperative discomfort than open surgery, leading to a quicker return to normal activities. The robotic system’s high-definition 3D view enhances precision and accuracy during the repair, which is especially advantageous for complex hernias and component separation procedures [34]. Comparative studies consistently show that robotic-assisted hernia repair results in shorter hospital stays, fewer complications, and faster recovery times than open repair. While the initial costs of robotic-assisted hernia repair are higher due to the expense of robotic platforms and specialized instruments, these costs can be offset over time by reduced hospital stays, fewer complications, and lower rates of readmissions and reoperations [35]. The adoption of robotic-assisted hernia repair has significantly increased in recent years, particularly for ventral and inguinal hernias. This trend is expected to continue as more surgeons gain experience with the technology and recognize its advantages. The evidence supporting robotic-assisted hernia repair underscores its benefits in providing shorter hospital stays, fewer complications, faster recovery, and improved outcomes for complex hernias, making it a valuable option for hernia repair [36].

Biological meshes and tissue engineering

Introduction to Biological Meshes

Biological meshes, also known as biologic meshes, are surgical implants composed of organic biomaterials such as porcine dermis, porcine small intestine submucosa, bovine dermis or pericardium, and human cadaveric dermis or fascia lata. These meshes are commonly employed in hernia repair, including inguinal and ventral hernias, as well as for hernia prophylaxis and contaminated hernia repairs. They are also utilized in pelvic floor dysfunction, parotidectomy, and reconstructive plastic surgery [37]. The main advantages of biological meshes include a reduced risk of infection compared to synthetic meshes and their ability to be absorbed into the scar tissue as part of cellular ingrowth. This absorption allows the mesh to integrate with the patient’s tissue, potentially decreasing the risk of chronic inflammation and foreign body reactions. However, biological meshes are generally more costly than synthetic options, and there is currently no comprehensive evidence to guide their optimal clinical application [10]. Biological meshes provide a collagen-rich scaffold that supports tissue remodeling and new collagen deposition. The rate of degradation and the mesh's ability to withstand mechanical stress can vary, with cross-linking being a factor that influences these characteristics. While cross-linking can enhance the mesh's structural integrity over time, it may also increase the risk of adhesion formation, which must be carefully considered [10]. Biological meshes offer potential benefits over synthetic meshes, particularly in reducing infection risk and promoting tissue integration. Nonetheless, more high-quality research is needed to fully understand their comparative effectiveness and safety across different surgical applications and to determine the optimal use cases and techniques for their implementation [10].

Mechanisms of Action and Advantages

The transabdominal retromuscular (TA-RM) approach for ventral hernia repair involves positioning the mesh in the retromuscular plane between the posterior rectus sheath and the rectus abdominis muscle. This technique ensures optimal mesh placement in an anatomical space while avoiding direct contact with abdominal viscera [38]. The eTEP approach is another minimally invasive method for ventral hernia repair. Like the TA-RM approach, eTEP places the mesh in the retromuscular plane via an extraperitoneal route, avoiding entry into the abdominal cavity. This helps to minimize complications related to mesh-bowel interaction [39]. A systematic review and meta-analysis comparing eTEP and TA-RM found no significant differences between the two techniques regarding major complications, surgical site infection rates, seroma formation, occurrences requiring procedural intervention, minor complications, intraoperative issues, recurrence, or postoperative ileus. Both approaches are deemed safe and effective minimally invasive options for ventral hernia repair [40]. Emerging techniques have introduced self-extendable mesh for minimally invasive repair of diaphragmatic hernias. This innovative device facilitates easier and more efficient mesh placement and fixation, potentially enhancing outcomes in complex cases [41]. Although laparoscopic and robotic IPOM repairs have been widely used, there are concerns about long-term mesh-related complications associated with intraperitoneal placement. Current guidelines recommend retromuscular mesh placement to mitigate these risks, increasing the adoption of extraperitoneal techniques such as eTEP and TA-RM [42].

Clinical Applications and Outcomes

Minimally invasive hernia repair techniques, including laparoscopic and robotic-assisted approaches, have gained prominence in recent years due to their numerous advantages over traditional open surgery. These methods offer smaller incisions, reduced scarring, less postoperative pain, and quicker recovery. Many patients can resume light activities within one to two weeks and return to strenuous activities after about a month. Additionally, minimally invasive techniques generally lower the risk of complications and infections [2]. Laparoscopic repair utilizes a camera and specialized instruments to visualize and repair the hernia through small incisions. Robotic-assisted repair follows a similar approach but enhances precision using robotic arms controlled by the surgeon. Minimally invasive IPOM repair is a reliable option for small to medium-sized hernias. Some studies have shown a low incidence of long-term mesh-related complications. Nonetheless, concerns about potential complications with intraperitoneal mesh placement have led to a preference for retromuscular placement in current guidelines [19]. While intraperitoneal mesh placement remains effective for certain cases, extraperitoneal techniques are often preferred to reduce the risk of mesh-related complications. For larger hernias, open repair with component separation may still be necessary. Overall, minimally invasive techniques have become a cornerstone of hernia treatment, offering significant benefits in reduced trauma, faster recovery, and lower complication rates compared to open surgery [43].

Emerging techniques and innovations

Role of Ultrasound in Hernia Diagnosis and Repair Planning

**Figure 1:**
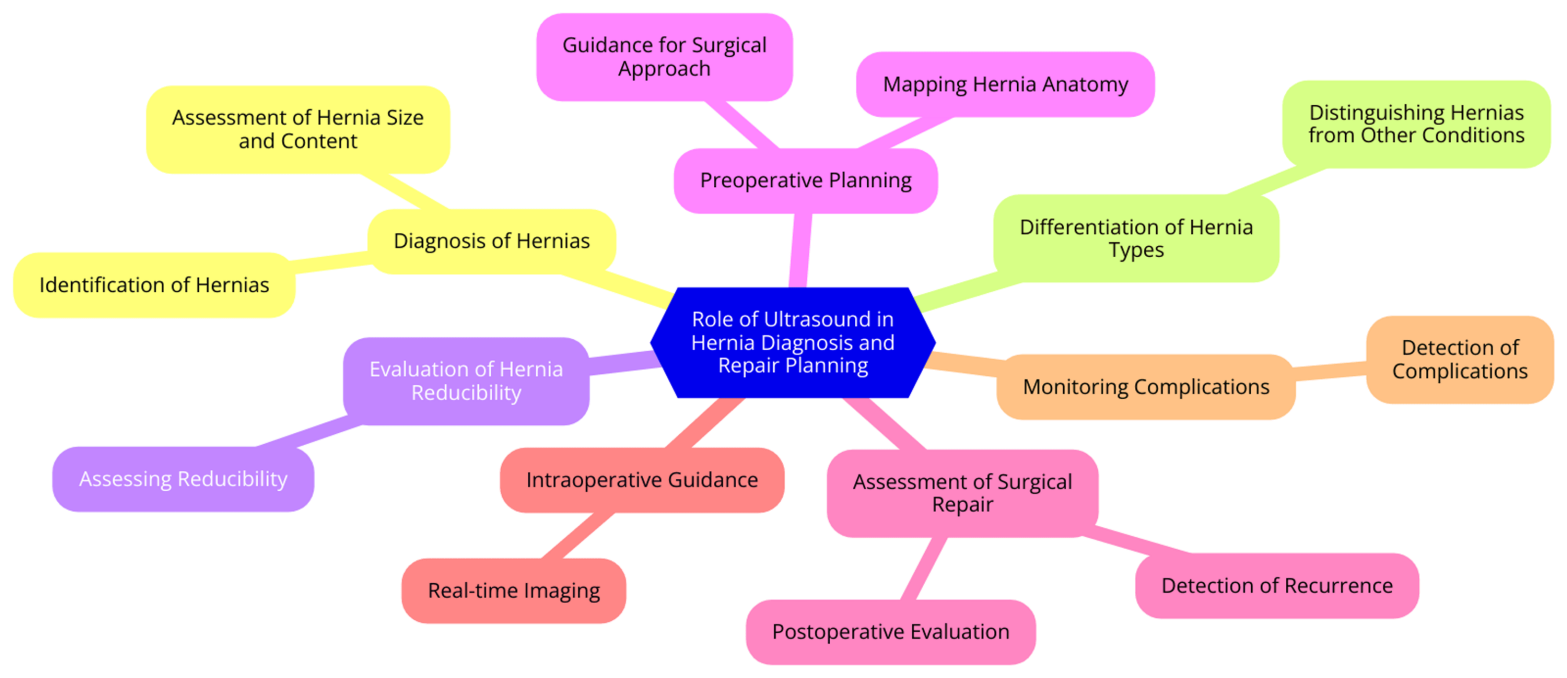
The role of ultrasound in hernia diagnosis and repair planning Image credit: Akansha Hatewar

The role of ultrasound in hernia diagnosis and repair planning has become increasingly crucial in modern surgical practice. Ultrasound has demonstrated high accuracy in diagnosing groin hernias, with studies reporting 100% sensitivity and 100% specificity. This imaging modality also differentiates between direct and indirect inguinal hernias, with sensitivity and specificity ranging from 86-97% [44]. In addition to its diagnostic accuracy, ultrasound offers several advantages over other imaging techniques. As a non-invasive, non-ionizing modality, it excels in soft tissue imaging of the groin and abdominal wall. Ultrasound is also more accessible and convenient than CT or magnetic resonance imaging (MRI), making it a practical choice for hernia evaluation [45]. Ultrasound's utility extends to hernia repair planning as well. Dynamic ultrasound imaging, which includes postural changes and the Valsalva maneuver, can help reproduce hernia symptoms and inform surgical planning. This real-time information aids surgeons in selecting the most effective approach and technique for repair [46]. Moreover, ultrasound is valuable for postoperative monitoring. It can detect complications and identify recurrence of hernias following surgical repair, facilitating timely intervention if necessary [46]. The role of ultrasound in hernia diagnosis and repair planning is shown in Figure 1.

3D Printing Applications in Customizing Hernia Meshes

3D printing is transforming the field of hernia repair by facilitating the creation of customized surgical meshes with advanced features. One notable application is the development of hernia meshes embedded with contrast agents such as barium, iodine, and gadolinium. These radio-opaque meshes can be 3D printed, which enhances their visibility on CT imaging after implantation. This innovation allows for the early detection of hernia recurrence or complications, leading to timely intervention and improved patient outcomes [47]. Another promising area of research involves the 3D printing of bioactive hernia meshes. Researchers are developing meshes with antimicrobial properties, drug delivery capabilities, and enhanced biocompatibility by integrating materials like polyurethane, alginate, and polycaprolactone with biological components. These bespoke 3D-printed meshes can potentially reduce infection risk, promote faster healing, and improve integration with the patient’s native tissues. The ability to tailor mesh properties to each patient's needs is a significant advantage of this technology [48]. In designing 3D-printed hernia meshes, it is essential to consider the biomechanical properties of the materials used. The mesh's strength, elasticity, and porosity must be precisely engineered to meet the specific demands of hernia repair. Emerging "4D printing" techniques, which utilize stimuli-responsive polymers, could enable 3D printed meshes to adapt dynamically to changes in the host tissue environment over time. Additionally, incorporating drug delivery capabilities into the mesh design could facilitate targeted release of therapeutics such as antibiotics or growth factors, further enhancing the mesh’s effectiveness and minimizing the risk of complications [48].

Future directions and ongoing research in hernia repair techniques

Hernia repair has seen significant advancements, particularly with the integration of minimally invasive techniques and innovative mesh materials. One notable development is introducing a novel self-extendable mesh device for diaphragmatic hernias. Recent studies have demonstrated that this device facilitates a safe and effective minimally invasive closure of these complex hernias, streamlining placement and fixation processes. This advancement represents a considerable improvement in managing congenital diaphragmatic hernias, offering better surgical outcomes and reducing procedural complexity [41]. Another area of innovation involves comparing minimally invasive approaches such as eTEP and TA-RM techniques for ventral hernia repair. A systematic review and meta-analysis have found no significant differences in major complications between these methods, suggesting that both offer comparable safety and efficacy. These techniques aim to extend the mesh into the optimal anatomical space while avoiding traumatic fixation, reflecting ongoing refinements in minimally invasive hernia repair strategies [40]. Addressing long-term mesh-related complications is also crucial in modern hernia repair. A long-term follow-up study of minimally invasive IPOM repairs has yielded reassuring results, indicating that this approach remains a safe and durable option for small to medium-sized hernias. The study reports a low incidence of long-term mesh-related complications, challenging concerns about catastrophic outcomes associated with IPOM repairs. This evidence suggests that the benefits of IPOM repairs may outweigh the associated risks [49].

## Conclusions

In conclusion, the field of hernia repair has witnessed remarkable advancements, transforming from traditional open surgeries with tension-free mesh repairs to sophisticated minimally invasive techniques that prioritize patient comfort and recovery. The evolution towards laparoscopic and robotic-assisted approaches has revolutionized surgical outcomes, offering reduced postoperative pain, shorter hospital stays, and quicker patient return to normal activities. Moreover, the introduction of biological meshes and ongoing innovations in 3D printing and ultrasound imaging continue to shape the future of hernia repair, promising further improvements in efficacy and patient satisfaction. As research and technology progress, clinicians must stay abreast of these developments to provide optimal care and enhance overall surgical outcomes in hernia repair.
